# Diffusion Tensor Imaging of the Sciatic Nerve as a Surrogate Marker for Nerve Functionality of the Upper and Lower Limb in Patients With Diabetes and Prediabetes

**DOI:** 10.3389/fnins.2021.642589

**Published:** 2021-03-03

**Authors:** Johann M. E. Jende, Zoltan Kender, Christoph Mooshage, Jan B. Groener, Lucia Alvarez-Ramos, Jennifer Kollmer, Alexander Juerchott, Artur Hahn, Sabine Heiland, Peter Nawroth, Martin Bendszus, Stefan Kopf, Felix T. Kurz

**Affiliations:** ^1^Department of Neuroradiology, Heidelberg University Hospital, Heidelberg, Germany; ^2^Department of Endocrinology, Diabetology and Clinical Chemistry, Heidelberg University Hospital, Heidelberg, Germany; ^3^Medicover Neuroendocrinology, Munich, Germany; ^4^German Center of Diabetes Research (DZD), Associated Partner in the DZD, München-Neuherberg, Germany; ^5^Division of Experimental Radiology, Department of Neuroradiology, Heidelberg, Germany; ^6^Joint Institute for Diabetes and Cancer at Helmholtz-Zentrum Munich and Heidelberg University, Heidelberg, Germany

**Keywords:** magnetic resonance imaging, diabetic polyneuropathy, magnetic resonance neurography, diffusion tensor imaging, fractional anisotropy, diabetes, prediabetes

## Abstract

**Background:**

Nerve damage in diabetic neuropathy (DN) is assumed to begin in the distal legs with a subsequent progression to hands and arms at later stages. In contrast, recent studies have found that lower limb nerve lesions in DN predominate at the proximal sciatic nerve and that, in the upper limb, nerve functions can be impaired at early stages of DN.

**Materials and Methods:**

In this prospective, single-center cross-sectional study, participants underwent diffusion-weighted 3 Tesla magnetic resonance neurography in order to calculate the sciatic nerve’s fractional anisotropy (FA), a surrogate parameter for structural nerve integrity. Results were correlated with clinical and electrophysiological assessments of the lower limb and an examination of hand function derived from the Purdue Pegboard Test.

**Results:**

Overall, 71 patients with diabetes, 11 patients with prediabetes and 25 age-matched control subjects took part in this study. In patients with diabetes, the sciatic nerve’s FA showed positive correlations with tibial and peroneal nerve conduction velocities (*r* = 0.62; *p* < 0.001 and *r* = 0.56; *p* < 0.001, respectively), and tibial and peroneal nerve compound motor action potentials (*r* = 0.62; *p* < 0.001 and *r* = 0.63; *p* < 0.001, respectively). Moreover, the sciatic nerve’s FA was correlated with the Pegboard Test results in patients with diabetes (*r* = 0.52; *p* < 0.001), prediabetes (*r* = 0.76; *p* < 0.001) and in controls (*r* = 0.79; *p* = 0.007).

**Conclusion:**

This study is the first to show that the sciatic nerve’s FA is a surrogate marker for functional and electrophysiological parameters of both upper and lower limbs in patients with diabetes and prediabetes, suggesting that nerve damage in these patients is not restricted to the level of the symptomatic limbs but rather affects the entire peripheral nervous system.

## Introduction

Distal symmetric diabetic polyneuropathy (DN) is one of the most severe complications of diabetes mellitus (Tesfaye et al., 2005; Feldman et al., 2017). DN is generally acknowledged to be a late complication of diabetes that starts at the level of the feet and then progresses further upwards until, at later stages, the upper limbs become involved as well, starting at the level of the hands (Nawroth et al., 2018). In contrast to the progression of clinical symptoms, recent studies applying high-resolution magnetic resonance neurography (MRN) at three Tesla (3T) have come to show that nerve lesions predominate proximally at the level of the sciatic nerve and that the sciatic nerve’s fractional anisotropy (FA), a dimensionless quantity for directed diffusion in nerve tissue, is a highly sensitive parameter for structural nerve damage in patients with diabetic neuropathy in previous clinical studies (Vaeggemose et al., 2017b; Jende et al., 2019, 2020a). Despite the assumption that length-dependent nerve damage in DN starts at the level of the feet and progresses to further proximally with an involvement of the upper limbs at later stages, recent studies revealed that sensory and motor functions of the upper limb are frequently affected already at early stages of DN but often remain undiagnosed until a certain degree of functional impairment becomes apparent, indicating that the progression of nerve fiber damage at the level of the hands and arms may parallel the progression of nerve fiber damage at the level of the feet and legs (Kopf et al., 2018a; Yang et al., 2018).

The aim of this study was to elucidate correlations of the sciatic nerve’s FA with clinical and electrophysiological parameters of the upper and lower limbs in patients with prediabetes and diabetes, and in an age-matched control group. Therefore, we chose an MRN protocol that combined T2-weighted and diffusion-weighted sequences with a subsequent automated approach for the calculation of the sciatic nerve’s FA.

## Materials and Methods

### Study Design and Participants

**FIGURE 1:**
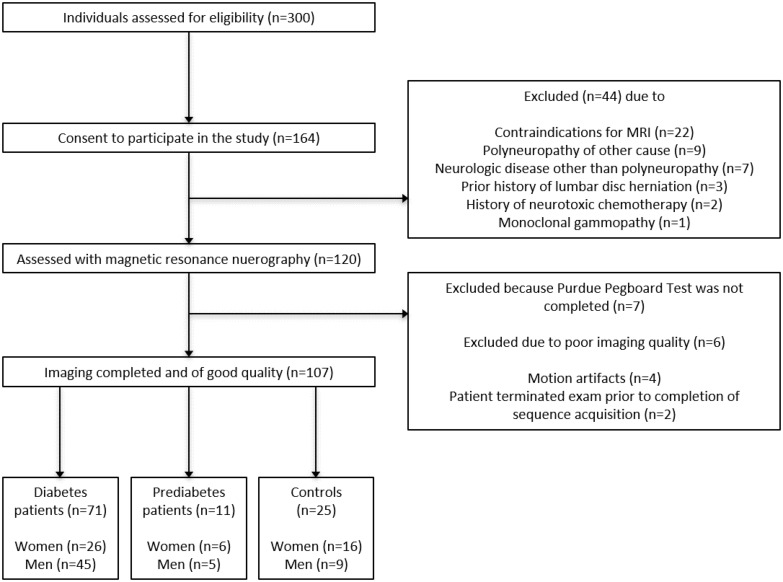
Process of patient recruitment.

The local ethics committee approved this study (HEIST-DiC, local ethics number S-383/2016^1^, identifier NCT03022721), and all study participants gave informed, written consent. Prediabetes was defined as fasting glucose levels of 100–125 md/dL or a 2-h glucose level of 140–199 mg/dL after 75 g glucose intake. Participants with glucose levels below these limits were defined as normal (healthy controls). Participants with glucose levels above the limit and absence of GAD or IA2 antibodies were defined as newly diagnosed type 2 diabetes (Kopf et al., 2018b). Overall, 71 patients with diabetes (12 with type 1 diabetes, 59 with type 2 diabetes; 26 female, 45 male; mean age 61.07 ± 1.57; age range 21-84 years), 11 patients with prediabetes (6 female, 5 male, 61.82 ± 2.85, age range 47-78) and 25 age-matched control subjects (16 female, 9 male, mean age 56.24 ± 2.17, age range 30-77) took part in this study between June 2016 and May 2020. Study participants were recruited from the Outpatient Clinic of Internal Medicine at Heidelberg University Hospital (Z.K., J.B.G., L.A., and S.K.). Participants formed a random series, meaning that they were not recruited and examined groupwise but in a randomized order. All participants were right-handed. Overall exclusion criteria were age <18, pregnancy, any history of lumbar surgery or disk extrusion, any contraindications for MR imaging, any other neuropathy-associated risk factors such as alcohol abuse, hypovitaminosis, malignant or infectious diseases, any previous or ongoing exposure to neurotoxic agents, monoclonal gammopathy, and any chronic neurological diseases such as Parkinson’s disease, multiple sclerosis, or restless legs syndrome. The process of patient recruitment is illustrated in Figure 1.

### Clinical and Electrophysiologic Examination

A detailed medical history was documented for every participant. The examination of neuropathic symptoms was performed according to the guidelines of the German Society for Diabetology comprising the neuropathy symptom score (NSS), the Neuropathy Disability Score (NDS) and electrophysiological examinations as outlined below (Young et al., 1993).

The electrophysiological examination (Viasys Healthcare VikingQuest^®^, Viasys Healthcare GmbH, Höchberg) of the right leg included: distal motor latencies of the right tibial and peroneal nerve, motor and sensory amplitudes (CMAPs and SNAPs, respectively) of the tibial, peroneal and sural nerves and nerve conduction velocities (NCVs) of the tibial, peroneal and sural nerves. It was assured that skin temperature was at least 32°C throughout the examination. Blood was drawn in fasting state and processed immediately under standardized conditions in the central laboratory of Heidelberg University Hospital. All examinations were performed by clinicians with expertise in diabetology and more than 8 years of clinical experience (Z.K., J.B.G., and S.K.) and were surveyed by a clinician with expertise in internal medicine, diabetology, and laboratory medicine (P.N.) with more than 30 years of experience.

### Measurement of Hand Dexterity and Fine Motoric Skills by Using the Purdue Pegboard Test

The Purdue Pegboard Test (Model 32020; Lafayette Instrument Co., Lafayette, IN, United States), an established and commonly used test for the assessment of both gross and fine motor skills of the arms and hands, was used to assess motoric skills and manual dexterity (Symonds et al., 2017). The test employs a board with two parallel rows of 25 cavities into which cylindrical metal pegs are to be placed and comprises a total of four trials. The test includes four subtests: testing of the dominant hand, testing of the non-dominant hand, testing of both hands, and an assembly test allowing use of both hands as described before. In the subsets for preferred, non-preferred, and both hands the examinee is required to place the pins as fast as possible, the score being the number of pins placed after 30 s (O’Donnell et al., 2017).

### MRN Imaging Protocol

We performed high-resolution MRN of the right thigh in a 3.0 Tesla MR-scanner (Magnetom TIM-TRIO, Siemens Healthineers, Erlangen, Germany) for all study participants, using a 15-channel transmit-receive extremity coil. Participants were put in a supine position on the MRI transport table with the legs facing the scanner opening; the leg imaging coil, a 15-channel transit-receive extremity coil, was placed around the right midthigh and mid lower leg. The following sequences were used:

(1) Axial high resolution T2-weighted turbo spin echo (TSE) 2D sequence with spectral fat saturation (mode: strong) without water suppression or magnetization preparation. The following parameters were used: relaxation time (TR) = 5,970 ms, echo time (TE) = 55 ms, field of view (FOV) = 160 mm^2^ × 160 mm^2^, matrix size = 512 × 512, slice thickness = 4 mm, interslice gap = 0.8 mm, voxel size = 0.3 mm^3^ × 0.3 mm^3^ × 4.0 mm^3^, 24 slices, 24 acquired images, receiver bandwidth = 181 Hz/pixel, echo spacing = 11.1 ms, turbo factor = 13, 15 echo trains per slice, parallel imaging factor = 2, averages = 3, acquisition time = 4:42 min.
(2) Axial diffusion-weighted 2-dimensional echo-planar sequence images were recorded with spectral attenuated inversion recovery fat suppression (saturation mode: skewed). The following parameters were used: TR = 5,100 ms; TE = 92.8 ms; b = 0 and 1,000 s/mm^2^; directions = 20; field of view 160 mm^2^ × 160 mm^2^; matrix size 128 × 128; axial fat-suppressed, diffusion-weighted 2-dimensional echo-planar sequence with the following parameters: TR = 5,100 ms; TE = 92.8 ms; b = 0 and 1,000 s/mm^2^; directions = 20; field of view = 160 mm^2^ × 160 mm^2^; matrix size = 128 × 128; slice thickness = 4 mm; voxel size = 1.3 mm^3^ × 1.3 mm^3^ × 4 mm^3^; no interslice gap, 24 slices, 1,512 acquired images, receiver bandwidth = 1,396 Hz/pixel, EPI factor = 128, parallel imaging factor = 3, averages = 3, acquisition time = 5:47 min.

MRN sequences were centered on the sciatic nerve’s bifurcation to ascertain that the anatomical region mapped by MRN was comparable in all participants. All MRN Studies were performed by radiologists with more than 5 years of clinical experiences in MRN studies (J.M.E.J., J.K., A.J., and F.T.K.).

### Image Post-processing and Analysis

The MRN analysis followed an established analysis pipeline for diffusion tensor imaging (DTI) images (Christidi et al., 2016). All images were pseudonymized and analyzed in an automated approach (J.M.E.J., C.M., F.T.K.) using Nordic BRAINEX, a Food and Drug Administration (FDA) approved processing software designed for automated calculation and reconstruction of fiber tracts in diffusion weighted imaging. Nordic BrainEx uses a variant of the Fiber Assignment by Continuous Tracking (FACT) algorithm to obtain fiber tracking within a volume-of-interest (VOI) (Mori et al., 1999; NordicNeuroLab AS, 2019). We closely followed the DTI module instructions for Nordic BRAINEX (NordicNeuroLab AS, 2019). DTI setting configurations were extracted from the headers of the uploaded DTI image data (e.g., number of gradient directions, averages, etc.). First, we co-registered DTI images to the T2w images. For subsequent pre-processing, we chose the options “motion correction” and “Eddy current correction” and adjusted a noise threshold to include all voxels within the sciatic nerve. We further chose a FA cutoff >0.1 as in previous studies on DTI analyses in peripheral nerves (Kwon et al., 2015; Oudeman et al., 2020) to reduce the impact of intra- and perineural fat on the calculated FA of fascicular structures (Kwon et al., 2015; Oudeman et al., 2020). The tract turning angle was chosen as 41.4 degrees, the minimum fiber length at 20 mm, and the number of seeds per voxel as 1, see also (NordicNeuroLab AS, 2019). We then manually focused a cube shaped VOI on the sciatic nerve.

**FIGURE 2:**
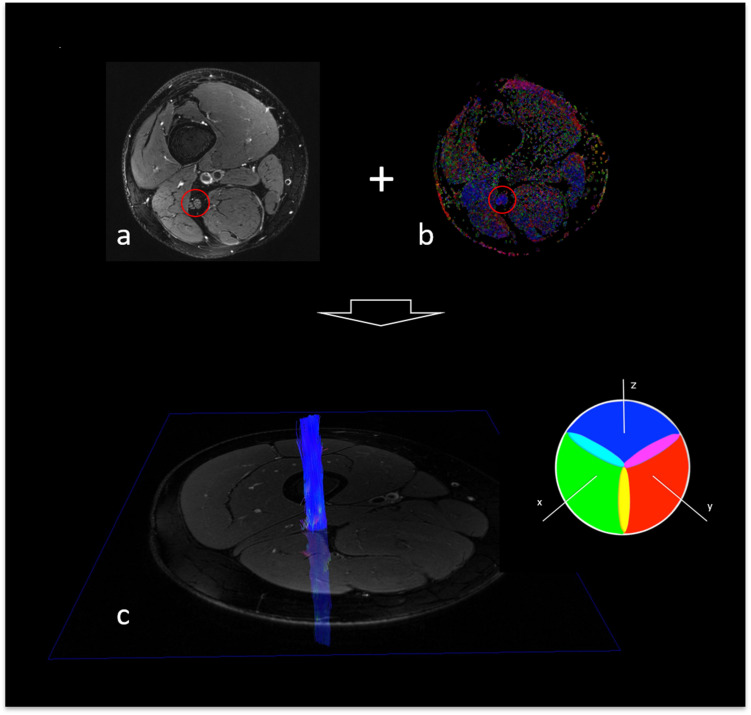
Fiber tracking of the sciatic nerve. **(a)** T2-weighted, fat suppressed image of the right thigh, depicting the sciatic nerve (red circle). **(b)** Colored map of fractional anisotropy (sciatic nerve encirceled in red). **(c)** Reconstructed, 3-dimensional fiber track of the right sciatic nerve with color encoding according to the DTI eigenvector color map where voxel color reflects the direction of the diffusion tensor in that voxel. Color intensity is scaled with the FA.

The tensor eigenvalues λ_1_, λ_2_, and λ_3_ and the average FA of the segmented nerve fibers were automatically determined in Nordic BRAINEX (NordicNeuroLab AS, 2019). Axial diffusivity (AD), radial diffusivity (RD) and mean diffusivity (MD) were calculated based on the obtained tensor eigenvalues as AD = λ_1_, RD = (λ_2_ + λ_3_)/2, and MD = (λ_1_ + λ_2_ + λ_3_)/3 ^16^. A graphic overview of the process of image co-registration and nerve segmentation is given in Figure 2.

### Statistical Analysis

Statistical data analysis was performed with GraphPad Prism 7 and Matlab v7.14.0.0739, R2012a (J.M.E.J., A.H., and F.T.K.). We tested for Gaussian normal distribution with the D’Agostino-Pearson omnibus normality test. Depending on Gaussian distribution, ANOVAs, or Kruskal-Wallis tests were performed for comparisons of three groups and Bonferoni-corrected Spearman or Pearson correlation coefficients for correlation analysis.

For all tests, the level of significance was defined at *p* < 0.05. All results are presented as mean values ± standard deviation.

## Results

### MRN Imaging, Demographic, Clinical and Serological Data

**FIGURE 3:**
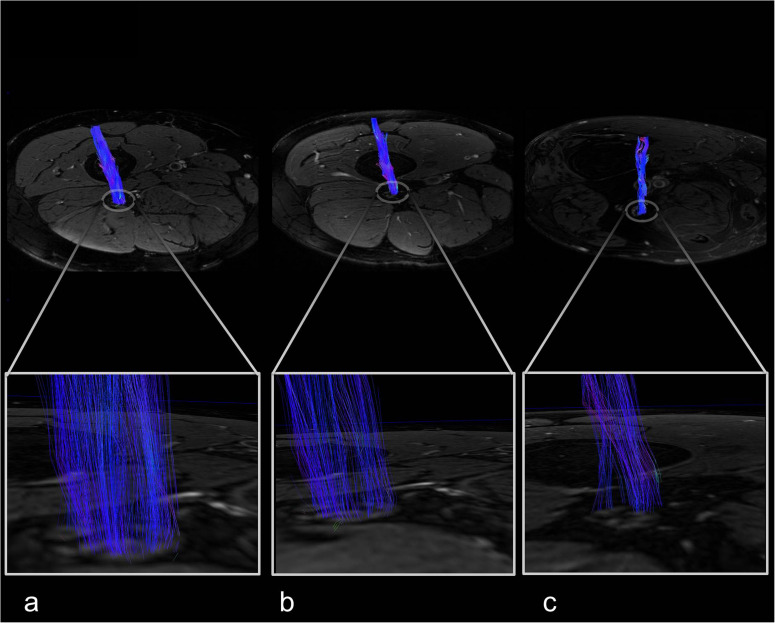
Sciatic nerve fiber tracts with magnified views. **(a)** *F*emale control, 67 years, neuropathy disability score (NDS) = 0. **(b)** *F*emale prediabetes patient, 57 years, NDS = 3. **(c)** Male type 2 diabetes patient, 58 years, NDS = 7.

There was no difference between the three groups for participants’ age (*p* = 0.231). HbA1c levels and triglyceride levels were higher in patients with diabetes when compared to patients with prediabetes or controls (*p* < 0.001 and *p* = 0.002, respectively). The total Pegboard score and the sciatic nerve’s FA were lower in patients with diabetes when compared to controls (*p* = 0.006). No such difference could be found for AD (*p* = 0.147), RD (*p* = 0.179), or MD (*p* = 0.810). In controls, patients with prediabetes and patients with diabetes, the total pegboard score was negatively correlated with age (*r* = −0.64; *p* = 0.001, *r* = 0.82; *p* = 0.002, and *r* = 0.60; *p* < 0.001, respectively). The NDS and the NSS score were higher in patients with diabetes when compared to controls (*p* < 0.001). The mean FA for controls (0.48 ± 0.06) and prediabetic patients (0.48 ± 0.08) was well within the reference range of a healthy group of volunteers [0.507 ± 0.05; (Kronlage et al., 2018)], whereas the mean FA for patients with diabetes was not (0.43 ± 0.08). An overview of participants’ characteristics is given in Table 1. Representative sciatic nerve fiber tracts from individuals of the three groups of participants (diabetes, prediabetes, controls) can be found in Figure 3. It can be seen that fiber tracts in the control patient are dense and contiguous (Figure 3a), which decreases for the prediabetes and diabetes patients (Figures 3b,c).

**TABLE 1:** Demographic, clinical, serological, and electrophysiological data of patients with diabetes, patients with prediabetes, and controls.

	Diabetes patients	Prediabetes patients	Controls	*p*
Age (years)	61.1 ± 13.5	61.8 ± 9.5	56.2 ± 10.9	0.231
Number of women	26	6	16	n.a.
Number of men	45	5	9	n.a.
Body mass index (kg/m^2^)	28.8 ± 4.1	28.5 ± 5.8	26.7 ± 6.4	0.131
NDS	3.0 ± 2.9	1.1 ± 1.7	0.9 ± 1.3	< 0.001
NSS	3.5 ± 3.3	1.9 ± 2.9	0.1 ± 0.6	< 0.001
Sciatic nerve’s FA	0.43 ± 0.08	0.48 ± 0.08	0.48 ± 0.06	0.006
Total purdue pegboard score	60.8 ± 11.8	65.3 ± 11.7	71.6 ± 11.2	< 0.001
HbA1c [% (mmol/mol)]	7.2 ± 1.28	5.7 ± 0.48	5.3 ± 0.36	< 0.001
	(55 ± 14)	(39 ± 5)	(34 ± 4)	
Cystatin C (mg/dl)	0.96 ± 0.25	0.98 ± 0.19	0.89 ± 0.14	0.451
GFR (ml/min)	88.6 ± 18.6	84.7 ± 17.3	89.9 ± 15.9	0.660
Triglycerides (mg/dl)	175.1 ± 187.0	145.4 ± 96.8	101.6 ± 44.0	0.002
Total Cholesterol (mg/dl)	188.7 ± 43.4	197.3 ± 41.8	209.0 ± 40.0	0.056
HDL Cholesterol	53.9 ± 19.1	54.4 ± 14.7	62.5 ± 19.0	0.033
LDL Cholesterol	104.2 ± 34.5	113.8 ± 41.2	126.2 ± 36.7	0.007
Sural NCV (m/s)	44.0 ± 11.5	45.0 ± 5.5	45.6 ± 8.6	0.882
Sural SNAP (μV)	6.3 ± 4.2	6.2 ± 3.5	8.4 ± 5.8	0.236
Peroneal NCV (m/s)	40.1 ± 8.2	42.6 ± 3.3	45.4 ± 3.9	0.002
Peroneal CMAP (μV)	6.1 ± 3.9	5.8 ± 3.2	8.6 ± 4.9	0.033
Tibial NCV (m/s)	40.3 ± 8.0	44.4 ± 2.2	46.5 ± 5.1	< 0.001
Tibial CMAP (μV)	11.3 ± 8.8	13.2 ± 6.2	15.4 ± 6.5	0.033

*p values are displayed as results from comparisons of the three groups either by ANOVA or Kruskal Wallis test, depending on the Gaussian or non-Gaussian distribution of data. All values are shown as mean ± standard deviation. n.a, not applicable; NDS, Neuropathy Disability Score; NSS, Neuropathy Severity Score; FA, fractional anisotropy; HbA1c, glycated hemoglobin; GFR, glomerular filtration rate; HDL, high density lipoprotein; LDL, low density lipoprotein; NCV, nerve conduction velocity; m/s, meters per second; SNAP, sensory nerve action potential; CMAP, compound motor action potential; μV, microvolt.*

A negative association was found between the sciatic nerves’ FA and patients’ age in patients with diabetes (*r* = −0.48; *p* < 0.001) and controls (*r* = −0.63; *p* = 0.001) but not in patients with prediabetes (*r* = 0.44; *p* = 0.202). FA was negatively correlated with the NDS score in patients with diabetes, (*r* = −0.53; *p* < 0.001) and controls (*r* = −0.47; *p* = 0.017). A similar, yet not significant trend was seen in patients with prediabetes (*r* = −0.58; *p* = 0.082). In patients with diabetes, further negative associations of the FA were found with cystatin C (*r* = −0.45; *p* < 0.001). A detailed survey of correlations between the sciatic nerve’s FA and demographic, clinical, and serological parameters is given in Table 2.

**TABLE 2:** Correlations of the sciatic nerve’s FA with clinical, epidemiological, and serological data of diabetes patients, prediabetes patients, and controls.

	FA diabetes patients		FA prediabetes patients		FA controls	
	*r*	*p*	*r*	*p*	*r*	*p*
Age (years)	–0.48	< 0.001	0.44	0.202	–0.63	0.001
Body mass index (kg/m^2^)	–0.18	0.140	–0.08	0.832	0.03	0.881
NDS	–0.53	< 0.001	–0.58	0.082	–0.47	0.017
NSS	–0.20	0.106	–0.57	0.107	–0.36	0.107
HbA1c (%)	0.15	0.211	–0.07	0.844	–0.42	0.039
Cystatin C (mg/l)	–0.45	< 0.001	–0.27	0.489	–0.22	0.320
Glomerular filtration rate (ml/min)	0.46	< 0.001	0.21	0.571	0.23	0.291
Triglycerides (mg/dl)	0.05	0.694	0.06	0.876	–0.04	0.867
Total serum cholesterol (mg/dl)	0.17	0.151	–0.10	0.776	–0.15	0.462
HDL cholesterol (mg/dl)	0.17	0.157	–0.11	0.762	0.07	0.728
LDL cholesterol (mg/dl)	0.10	0.414	0.05	0.770	–0.18	0.396
Sural NCV(m/s)	0.24	0.128	–0.28	0.505	–0.12	0.568
Sural SNAP (μV)	0.47	0.006	0.30	0.471	0.58	0.003
Tibial NCV (m/s)	0.47	< 0.001	0.61	0.081	0.35	0.099
Tibial CMAP (μV)	0.62	< 0.001	0.61	0.081	0.30	0.150
Peroneal NCV (m/s)	0.52	< 0.001	0.43	0.246	0.31	0.142
Peroneal CMAP (μV)	0.62	< 0.001	0.51	0.160	0.18	0.411
Total Pegboard Test score	0.52	< 0.001	0.79	0.007	0.76	< 0.001
Pegboard Test of dominant hand	0.61	< 0.001	0.65	0.044	0.64	0.001
Pegboard Test of non-dominant hand	0.43	< 0.001	0.71	0.022	0.55	0.005
Pegboard Test of both hands	0.38	0.002	0.70	0.024	0.59	0.002
Pegboard Assembly Test	0.46	< 0.001	0.71	0.022	0.61	0.001

*NDS, Neuropathy Disability Score; NSS, Neuropathy Severity Score; HbA1c, glycated hemoglobin; GFR, glomerular filtration rate; HDL, high density lipoprotein; LDL, low density lipoprotein; NCV, nerve conduction velocity; m/s, meters per second; SNAP, sensory nerve action potential; CMAP, compound motor action potential; μV, microvolt.*

Similarly, as in previous studies, (Vaeggemose et al., 2017a, 2020; Kronlage et al., 2018). RD and MD (or apparent diffusion coefficient ADC) showed correlations with clinical parameters that are in line with the correlations obtained for the fractional anisotropy FA. For instance, the mean diffusivity in the diabetes cohort is highly correlated with tibial NCV (*r* = −0.31, *p* = 0.004), and the Pegboard of the dominant and non-dominant hand (*r* = −0.26, *p* = 0.026, and *r* = −0.31, *p* = 0.008, respectively), as is the FA (*r* = 0.47, *p* < 0.001, and *r* = 0.61, *p* < 0.001, and *r* = 0.43, *p* < 0.001, respectively). Correlations of FA values with clinical, epidemiological, and serological data were generally better than those of RD and MD, which is why we focused on FA values; however, correlations of AD, RD, MD, and DTI tensor eigenvalues λ_2_ and λ_3_ with all relevant parameters are given in the Supplementary Tables 1-5, respectively.

### MRN Results and Electrophysiological Data of the Lower Limb

In patients with diabetes, the sciatic nerves’ FA showed positive correlations with tibial and peroneal NCVs (*r* = 0.47; *p* < 0.001, *r* = 0.52; *p* < 0.001, respectively), with tibial and peroneal nerve compound muscle action potential (CMAP) amplitudes and sural nerve sensory nerve action potential (SNAP) amplitudes (*r* = 0.62; *p* < 0.001, *r* = 0.62; *p* < 0.001, and *r* = 0.47; *p* = 0.006, respectively). A similar, yet not significant trend was found for tibial nerve NCV and CMAP amplitudes in patients with prediabetes (*r* = 0.61; *p* = 0.081 and *r* = 0.61; *p* = 0.081, respectively). A summary of all correlations between the FA and electrophysiological data of the lower limb is given in Table 2.

### MRN Results and Hand Function

**FIGURE 4:**
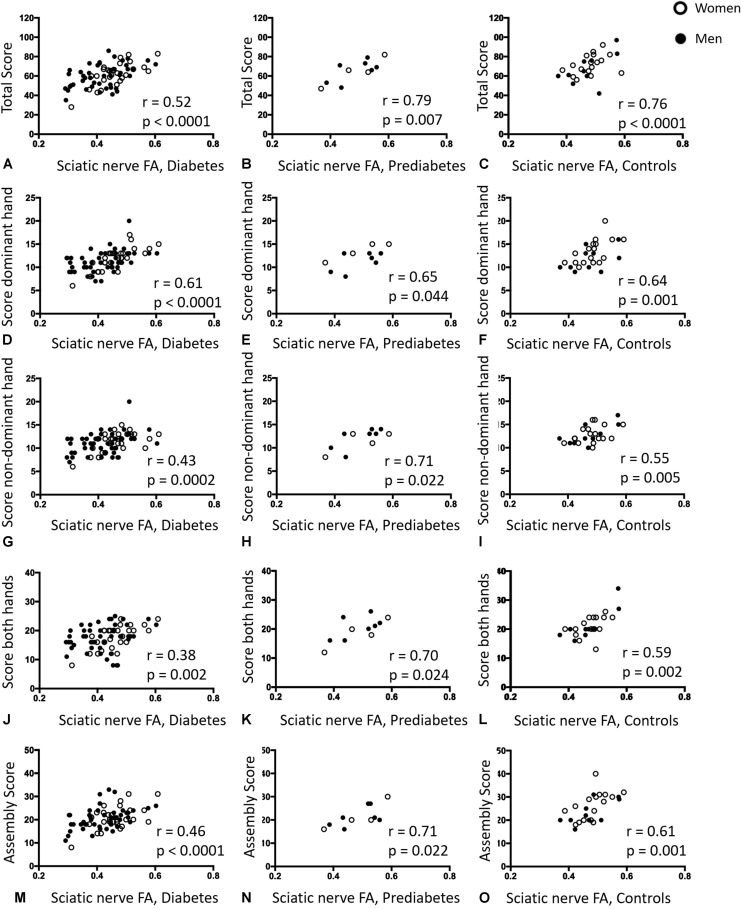
Correlative statistics of Purdue Pegboard Test scores and sciatic nerve fractional anisotropy. **(A)** Correlation of the sciatic nerve’s fractional anisotropy (FA) in patients with diabetes with the total Purdue Pegboard Test score (*r* = 0.52; *p* < 0.001; 95% confidence interval (CI) = 0.31 to 0.67). **(B)** Correlation of the sciatic nerve’s FA in patients with prediabetes with the total Purdue Pegboard Test score (*r* = 0.79; *p* = 0.007; 95% CI = 0.31 to 0.95). **(C)** Correlation of the sciatic nerve’s FA in controls with the total Purdue Pegboard Test score (*r* = 0.76; *p* < 0.001; 95%CI = 0.52 to 0.89). **(D)** Correlation of the sciatic nerve’s FA in patients with diabetes with the Purdue Pegboard Test score of the dominant hand (*r* = 0.61; *p* < 0.001; 95%CI = 0.43 to 0.74). **(E)** Correlation of the sciatic nerve’s FA in patients with prediabetes with the Purdue Pegboard Test score of the dominant hand (*r* = 0.65; *p* = 0.044; 95%CI = 0.52 to 0.90). **(F)** Correlation of the sciatic nerve’s FA in controls with the Purdue Pegboard Test score of the dominant hand (*r* = 0.64; *p* = 0.001; 95%CI = 0.22 to 0.77). **(G)** Correlation of the sciatic nerve’s FA in patients with diabetes with the Purdue Pegboard Test score of the non-dominant hand (*r* = 0.43; *p* = 0.0002; 95%CI = 0.22 to 0.60). **(H)** Correlation of the sciatic nerve’s FA in patients with prediabetes with the Purdue Pegboard Test score of the non-dominant hand (*r* = 0.71; *p* = 0.022; 95%CI = 0.21 to 0.92). **(I)** Correlation of the sciatic nerve’s FA in controls with the Purdue Pegboard Test score of the non-dominant hand (*r* = 0.55; *p* = 0.005; 95%CI = 0.19 to 0.76). **(J)** Correlation of the sciatic nerve’s FA in patients with diabetes with the Purdue Pegboard Test score of both hands (*r* = 0.38; *p* = 0.002; C95%CI = 0.14 to 0.54). **(K)** Correlation of the sciatic nerve’s FA in patients with prediabetes with the Purdue Pegboard Test score of both hands (*r* = 0.70; *p* = 0.024; 95%CI = 0.16 to 0.91). **(L)** Correlation of the sciatic nerve’s FA in controls with the Purdue Pegboard Test score of both hands (*r* = 0.59; *p* = 0.002; 95%CI = 0.37 to 0.84). **(M)** Correlation of the sciatic nerve’s FA in patients with diabetes with the Purdue Pegboard Test assembly score (*r* = 0.46; *p* < 0.001; 95%CI = 0.26 to 0.63). **(N)** Correlation of the sciatic nerve’s FA in patients with prediabetes with the Purdue Pegboard Test assembly score (*r* = 0.71; *p* = 0.022; 95%CI = 0.20 to 0.92). **(O)** Correlation of the sciatic nerve’s FA in controls with the Purdue Pegboard Test assembly score (*r* = 0.61; *p* = 0.001; 95%CI = 0.26 to 0.79).

The sciatic nerves’ FA correlated positively with the total score of the pegboard-test in patients with diabetes, patients with prediabetes and controls (*r* = 0.52; *p* < 0.001, *r* = 0.79; *p* = 0.007, and *r* = 0.76; *p* < 0.001, respectively, Figures 4A-C). In all 4 subtests that comprise the Pegboard test, this finding could be reproduced: for patients with diabetes, patients with prediabetes and controls, significant correlations were found between FA and Pegboard scores of the dominant hand (Figures 4D-F) non-dominant hand (Figures 4G-I), testing of both hands (Figures 4J-L), and the assembly test (Figures 4M-O). A summary of the correlations between the sciatic nerves’ FA and results of the Purdue Pegboard test is given in Table 2.

Corresponding correlations for the subgroups of men and women are clearly visible, especially for the subgroup of women. We also tabularized the results from correlation analyses in both subgroups in Supplementary Table 6. Except for the Pegboard test of both hands in the men subgroup, all Pegboard parameters were significantly correlated with FA in the diabetes group. There was also a mild correlation of the Pegboard test of the dominant hand in the women subgroup and the control subgroup, as well as some further significant correlations between Pegboard parameters and FA in the control subgroups. Missing correlations in the prediabetes subgroups may be due to the small number of patients.

## Discussion

To our knowledge, this study is the first to show that the sciatic nerve’s FA as a surrogate marker for nerve integrity correlates both with electrophysiological parameters of nerves in the distal lower limb and parameters of hand function in patients with diabetes. Also, this study is the first to show correlations between the sciatic nerve’s FA and parameters of hand function in patients with prediabetes. Both results are of importance with regards to understanding the course of nerve damage in diabetic neuropathy: despite the assumption that nerve damage in DN parallels clinical symptoms and progresses from distally to proximally starting at the level of the feet and progressing further onward to the level of the hands at later stages, our results indicate that neuropathic changes at the level of the upper limbs parallel those at the level of the lower limbs.

The finding that an association between the sciatic nerve’s FA and functional parameters of the hands and the lower leg can already be displayed in patients with prediabetes suggests that functional impairment of the upper extremities occurs during the development of pathological glucose tolerance that later progresses into type 2 diabetes (American Diabetes Association, 2017). This is of importance for the understanding of the progression of neuropathic changes leading to diabetic neuropathy, since these findings suggest that structural nerve damage related to functional impairment occurs prior to or at very early stages of type 2 diabetes and is not, as mostly assumed, a late complication of this disease (Jende et al., 2018, 2020c). The finding that the proximally located sciatic nerve’s FA correlates with functional parameters of the distal upper and lower limbs further indicates that the deterioration of nerve microstructure does not progress from distally to further proximally but rather suggests that the entire peripheral nervous system is already affected at the very beginning of DN. This is in line with results from previous studies on MRN in patients with and without DN that have found a proximal predominance of nerve lesions in DN in T2-weighted imaging (Jende et al., 2018, 2020b).

Since studies on MRN have come to show that nerve damage predominates at a proximal level in various neuropathies, the finding that the sciatic nerve’s FA codifies functional parameters of the upper limb not just in patients with prediabetes and diabetes but also in controls without impaired glucose tolerance, may be of relevance not just for the course of nerve damage in DN but also other diseases of the central and peripheral nervous system (Jende et al., 2017). Additionally, our findings suggest that FA values represent an objective parameter for assessing the structural integrity of the entire peripheral nervous system in longitudinal clinical studies or interventional studies in patients with DN. One may of curse argue that, since there was a correlation between participants’ age and the sciatic nerve’s FA in controls and patients with diabetes, the results of this study only display the well-established effect of aging on the structural and functional integrity of the peripheral nervous system (Kronlage et al., 2018). It should be considered, however, that in patients with prediabetes there was no correlation between age and FA whereas correlations between FA and parameters of hand function were significant.

The finding that there is no correlation between FA and patients’ age in prediabetes patients but a positive correlation between FA and the scores of the Pegboard test can be explained by the fact that the performance in the Purdue Pegboard test is not just a matter of peripheral nerve function but also depends on cognitive and coordinative skills, which are known to decline with age (Geffe et al., 2016). The finding that there is a correlation of the Pegboard test’s results with structural nerve integrity represented by the sciatic nerve’s FA indicates that a reduction of the latter is associated with a reduced performance in the Pegboard test and that, therefore, the structural integrity of peripheral nerves is of critical relevance for the performance in coordinative tasks. The finding that a reduction in FA is not correlated with age in the prediabetes group indicates that, other than cognitive and motor skills at the level of the central nervous system, diabetes-related structural damage to peripheral nerves occurs at very early stages of this disorder. This hypothesis is further supported by the finding that, while participant groups were matched for age, FA was significantly lower in diabetes patients when compared to controls. This indicates that, in addition to the process of aging, metabolic changes associated with prediabetes and diabetes cause auxiliary structural damage to peripheral nerves (Groener et al., 2020). However, it should be noted further that correlations between FA and Pegboard test parameters were significantly weaker for patients in the prediabetes group than for diabetes patients or the control group. Similarly, there are no significant correlations in the prediabetes group between FA and sural SNAP parameters or age, while there are in the diabetes and control group. In order to determine the exact impact of age and metabolic factors on nerve damage in prediabetes more closely, longitudinal studies with larger cohorts of prediabetes patients are required.

One may also argue that this study did not differentiate between type 1 diabetes and type 2 diabetes patients, although previous studies have shown that structural nerve damage differs between diabetes types for T2-weighted imaging (Jende et al., 2018). For DTI imaging, however, FA was found as a reliable marker for structural nerve integrity at the level of the lower limbs in both type 1 and type 2 diabetes (Vaeggemose et al., 2017a, 2020).

Our study is limited by the fact that only cross-sectional data were used, which does not allow for conclusions on the predictive value of the sciatic nerve’s FA. Also, the group of prediabetes patients was too small to allow for exact conclusions of the interaction between aging and metabolic factors. Another limitation is that we were only testing motor function and coordination of the upper limbs but did not test for sensory gain or loss.

While, usually, improved tractography methods to select multi fiber tracts in cerebral imaging can be challenging due to the difficulty of selecting adequate regions of interest for tract seeding (Wilkins et al., 2015; Christidi et al., 2016), the VOI selection in sciatic nerve processing is straight-forward due to the nature of peripheral nerves that can be easily identified on T2w images. Nordic BrainEx was shown to agree in major fiber bundle reconstruction with other major software vendors such as DSI Studio or Philips FiberTrak, while there were differences for smaller fiber bundles (Christidi et al., 2016). It should therefore be noted that systematic errors during the automated reconstruction process may impact the DTI parameter results, however, the FACT algorithm used in Nordic BRAINEX is a commonly used DTI reconstruction algorithm, and seeding errors are supposedly low in lieu of the above considerations (Mukherjee et al., 2008). Further potential sources of error are the FA cutoff, which we chose in agreement with previous publications on peripheral nerve DTI analyses (Kwon et al., 2015; Oudeman et al., 2020), partial volume effects, and, naturally, experimental parameters such as resolution and signal-to-noise ratios (Okamoto et al., 2010; Barrio-Arranz et al., 2015). We chose a comparatively high DTI in-plane resolution, see e.g. (van Steenkiste et al., 2016), to reduce DTI processing errors, as well as multiple averages to reduce signal-to-noise ratio. Considering that sciatic nerve fat fractions are elevated in patients with increased BMI, a missing correlation of sciatic nerve FA with BMI (Table 2) indicates that there is no significant impact of intra- or perineural fat on FA calculation.

One further limitation may be the spatial heterogeneity of the gradients (Krzyżak and Olejniczak, 2015; Borkowski and Krzyżak, 2019). Taken together, these types of errors are likely averaged for a larger number of patients, as in the diabetes and control group.

In summary, this study is the first to show that the sciatic nerve’s FA is a surrogate marker for nerve function of the distal upper and lower limbs in patients with diabetes and prediabetes. Our findings suggest that proximal nerve damage in diabetes parallels distal nerve function even before patients start to experience clinical symptoms, which may also be relevant with regard to PNS damage in other neuropathies. Further longitudinal studies on the predictive value of the sciatic nerve’s FA for disease progression in DN and other neuropathies are warranted.

## Data Availability Statement

The datasets presented in this article are not readily available because they contain sensitive patient information. The data supporting the conclusions of this article will be made available upon reasonable request by any qualified researcher. Requests to access the datasets should be directed to FK, felix.kurz@med.uni-heidelberg.de.

## Ethics Statement

The studies involving human participants were reviewed and approved by the Ethics Committee of the Medical Faculty of the University of Heidelberg. The patients/participants provided their written informed consent to participate in this study.

## Author Contributions

JJ, MB, SH, PN, and FK designed and coordinated the study. JJ, CM, JG, JK, AJ, and FK contributed to the organization of participants. JJ, AJ, and FK collected MR data. AH and FK developed image analysis tools. ZK, LA-R, JG, and SK collected clinical, serological, and electrophysiological data. JJ and FK analyzed the data, wrote the manuscript with input from all co-authors.

## Conflict of Interest

The authors declare that the research was conducted in the absence of any commercial or financial relationships that could be construed as a potential conflict of interest.

## Footnotes

1 clinicaltrials.gov

## Abbreviations

CI: confidence interval
CMAP: compound motor action potential
DN: diabetic neuropathy
mV: microvolt
NCV: nerve conduction velocity
NDS: neuropathy disability score
NSS: neuropathy severity score
SNAP: sensory nerve action potential.

## References

[B1] American Diabetes Association (2017). 2. Classification and diagnosis of diabetes. *Diabetes Care* 40(Suppl. 1) S11–S24. 10.2337/dc17-S005 27979889

[B2] Barrio-Arranz G. de Luis-García R. Tristán-Vega A. Martín-Fernández M. Aja-Fernández S. (2015). Impact of MR acquisition parameters on DTI scalar indexes: a tractography based approach. *PLoS One* 10:e0137905. 10.1371/journal.pone.0137905 26457415 PMC4601730

[B3] Borkowski K. Krzyżak A. T. (2019). Assessment of the systematic errors caused by diffusion gradient inhomogeneity in DTI-computer simulations. *NMR Biomed.* 32:e4130. 10.1002/nbm.4130 31343807

[B4] Christidi F. Karavasilis E. Samiotis K. Bisdas S. Papanikolaou N. (2016). Fiber tracking: a qualitative and quantitative comparison between four different software tools on the reconstruction of major white matter tracts. *Eur. J. Radiol. Open* 3 153–161. 10.1016/J.EJRO.2016.06.002 27489869 PMC4959946

[B5] Feldman E. L. Nave K.-A. Jensen T. S. Bennett D. L. H. (2017). New horizons in diabetic neuropathy: mechanisms, bioenergetics, and pain. *Neuron* 93 1296–1313. 28334605 10.1016/j.neuron.2017.02.005 PMC5400015

[B6] Geffe S. Schindlbeck K. A. Mehl A. Jende J. Klostermann F. Marzinzik F. (2016). The single intake of levodopa modulates implicit learning in drug naïve, de novo patients with idiopathic Parkinson’s disease. *J. Neural Transm.* 123 601–610. 10.1007/s00702-016-1557-y 27106907

[B7] Groener J. B. Jende J. M. E. Kurz F. T. Kender Z. Treede R.-D. Schuh-Hofer S. (2020). Understanding diabetic neuropathy-from subclinical nerve lesions to severe nerve fiber deficits: a cross-sectional study in patients with type 2 diabetes and healthy control subjects. *Diabetes* 69 436–447. 10.2337/db19-0197 31826867

[B8] Jende J. M. E. Groener J. B. Kender Z. Hahn A. Morgenstern J. Heiland S. (2020a). Troponin T parallels structural nerve damage in type 2 diabetes: a cross-sectional study using magnetic resonance neurography. *Diabetes* 69 713–723. 10.2337/db19-1094 31974140

[B9] Jende J. M. E. Groener J. B. Kender Z. Rother C. Hahn A. Hilgenfeld T. (2020b). Structural nerve remodeling at 3-T MR neurography differs between painful and painless diabetic polyneuropathy in type 1 or 2 diabetes. *Radiology* 294 405–414. 10.1148/radiol.2019191347 31891321

[B10] Jende J. M. E. Groener J. B. Oikonomou D. Heiland S. Kopf S. Pham M. (2018). Diabetic neuropathy differs between type 1 and type 2 diabetes: insights from magnetic resonance neurography. *Ann. Neurol.* 83 588–598. 29443416 10.1002/ana.25182

[B11] Jende J. M. E. Groener J. B. Rother C. Kender Z. Hahn A. Hilgenfeld T. (2019). Association of serum cholesterol levels with peripheral nerve damage in patients with type 2 diabetes. *JAMA Netw. Open* 2:e194798. 10.1001/jamanetworkopen.2019.4798 PMC6547108 31150078

[B12] Jende J. M. E. Hauck G. H. Diem R. Weiler M. Heiland S. Wildemann B. (2017). Peripheral nerve involvement in multiple sclerosis: demonstration by magnetic resonance neurography. *Ann. Neurol.* 82 676–685. 29023976 10.1002/ana.25068

[B13] Jende J. M. E. Kender Z. Rother C. Alvarez-Ramos L. Groener J. B. Pham M. (2020c). Diabetic polyneuropathy is associated with pathomorphological changes in human dorsal root ganglia: a study using 3T MR neurography. *Front. Neurosci.* 14:570744. 10.3389/fnins.2020.570744 33100960 PMC7546893

[B14] Kopf S. Groener J. B. Kender Z. Fleming T. Bischoff S. Jende J. (2018a). Deep phenotyping neuropathy: an underestimated complication in patients with pre-diabetes and type 2 diabetes associated with albuminuria. *Diabetes Res. Clin. Pract.* 146 191–201. 10.1016/j.diabres.2018.10.020 30389624

[B15] Kopf S. Groener J. B. Kender Z. Fleming T. Brune M. Riedinger C. (2018b). Breathlessness and restrictive lung disease: an important diabetes-related feature in patients with type 2 diabetes. *Respiration* 96 29–40. 10.1159/000488909 29874679

[B16] Kronlage M. Schwehr V. Schwarz D. Godel T. Uhlmann L. Heiland S. (2018). Peripheral nerve diffusion tensor imaging (DTI): normal values and demographic determinants in a cohort of 60 healthy individuals. *Eur. Radiol.* 28 1801–1808. 10.1007/s00330-017-5134-z 29230526

[B17] Krzyżak A. T. Olejniczak Z. (2015). Improving the accuracy of PGSE DTI experiments using the spatial distribution of b matrix. *Magn. Reson. Imaging* 33 286–295. 10.1016/j.mri.2014.10.007 25460327

[B18] Kwon B. C. Koh S. H. Hwang S. Y. (2015). Optimal parameters and location for diffusion-tensor imaging in the diagnosis of carpal tunnel syndrome: a prospective matched case-control study. *AJR Am. J. Roentgenol.* 204 1248–1254. 10.2214/AJR.14.13371 26001235

[B19] Mori S. Crain B. J. Chacko V. P. van Zijl P. C. (1999). Three-dimensional tracking of axonal projections in the brain by magnetic resonance imaging. *Ann. Neurol.* 45 265–269. 10.1002/1531-8249(199902)45:2<265::aid-ana21<3.0.co;2-3 9989633

[B20] Mukherjee P. Berman J. I. Chung S. W. Hess C. P. Henry R. G. (2008). Diffusion tensor MR imaging and fiber tractography: theoretic underpinnings. *Am. J. Neuroradiol.* 29 632–641. 10.3174/ajnr.A1051 18339720 PMC7978191

[B21] Nawroth P. P. Bendszus M. Pham M. Jende J. Heiland S. Ries S. (2018). The quest for more research on painful diabetic neuropathy. *Neuroscience* 387 28–37. 10.1016/j.neuroscience.2017.09.023 28942323

[B22] NordicNeuroLab AS (2019). *nordic BrainEX Tutorial - DTI Module.* Available online at: http://downloads.nordicneurolab.com/SW/nBX/Tutorials/Tutorial%20-%20DTI.pdf (accessed January 31, 2021).

[B23] O’Donnell L. J. Suter Y. Rigolo L. Kahali P. Zhang F. Norton I. (2017). Automated white matter fiber tract identification in patients with brain tumors. *Neuroimage Clin.* 13 138–153. 10.1016/j.nicl.2016.11.023 27981029 PMC5144756

[B24] Okamoto Y. Kunimatsu A. Kono T. Nasu K. Sonobe J. Minami M. (2010). Changes in MR diffusion properties during active muscle contraction in the calf. *Magn. Reson. Med. Sci.* 9 1–8. 10.2463/mrms.9.1 20339260

[B25] Oudeman J. Eftimov F. Strijkers G. J. Schneiders J. J. Roosendaal S. D. Engbersen M. P. (2020). Diagnostic accuracy of MRI and ultrasound in chronic immune-mediated neuropathies. *Neurology* 94 e62–e74. 10.1212/WNL.0000000000008697 31827006

[B26] Symonds T. Campbell P. Randall J. A. (2017). A review of muscle- and performance-based assessment instruments in DM1. *Muscle Nerve* 56 78–85. 10.1002/mus.25468 27862025

[B27] Tesfaye S. Chaturvedi N. Eaton S. E. M. Ward J. D. Manes C. Ionescu-Tirgoviste C. (2005). Vascular risk factors and diabetic neuropathy. *N. Engl. J. Med.* 352 341–350. 10.1056/NEJMoa032782 15673800

[B28] Vaeggemose M. Haakma W. Pham M. Ringgaard S. Tankisi H. Ejskjaer N. (2020). Diffusion tensor imaging MR neurography detects polyneuropathy in type 2 diabetes. *J. Diabetes Complications* 34:107439. 10.1016/j.jdiacomp.2019.107439 31672457

[B29] Vaeggemose M. Pham M. Ringgaard S. Tankisi H. Ejskjaer N. Heiland S. (2017a). Diffusion tensor imaging MR neurography for the detection of polyneuropathy in type 1 diabetes. *J. Magn. Reson. Imaging* 45 1125–1134. 10.1002/jmri.25415 27472827

[B30] Vaeggemose M. Pham M. Ringgaard S. Tankisi H. Ejskjaer N. Heiland S. (2017b). Magnetic resonance neurography visualizes abnormalities in sciatic and tibial nerves in patients with type 1 diabetes and neuropathy. *Diabetes* 66 1779–1788. 10.2337/db16-1049 28432188

[B31] van Steenkiste G. Jeurissen B. Baete S. den Dekker A. J. Poot D. H. J. Boada F. (2016). *High Resolution Diffusion Tensor Reconstruction from Simultaneous Multi-Slice Acquisitions in a Clinically Feasible Scan time. Proceedings of the International Society for Magnetic Resonance in Medicine* 0002. Available online at: http://ismrm.gitlab.io/2016/0002.html (Accessed February 3, 2021).

[B32] Wilkins B. Lee N. Gajawelli N. Law M. Leporé N. (2015). Fiber estimation and tractography in diffusion MRI: development of simulated brain images and comparison of multi-fiber analysis methods at clinical b-values. *Neuroimage* 109 341–356. 10.1016/j.neuroimage.2014.12.060 25555998 PMC4600612

[B33] Yang C.-J. Hsu H.-Y. Lu C.-H. Chao Y.-L. Chiu H.-Y. Kuo L.-C. (2018). Do we underestimate influences of diabetic mononeuropathy or polyneuropathy on hand functional performance and life quality? *J. Diabetes Investig.* 9 179–185. 10.1111/jdi.12649 28267271 PMC5754520

[B34] Young M. J. Boulton A. J. MacLeod A. F. Williams D. R. Sonksen P. H. (1993). A multicentre study of the prevalence of diabetic peripheral neuropathy in the United Kingdom hospital clinic population. *Diabetologia* 36 150–154. 10.1007/BF00400697 8458529

