# Supplementary material for: Diffusion Tensor Imaging of the Sciatic Nerve as a Surrogate Marker for Nerve Functionality of the Upper and Lower Limb in Patients With Diabetes and Prediabetes
Source: Front Neurosci. 2021 Mar 3;15:642589. doi: 10.3389/fnins.2021.642589 (PMC7966816; doi:10.3389/fnins.2021.642589)
Supplement: Supplementary file 1 [file Data_Sheet_1.PDF]

Supplementary material, Table S1

Table S1: Correlations of the sciatic nerve's DTI axial diffusivity (AD) with clinical, epidemiological, and serological data of diabetes patients, prediabetes patients, and controls.

	AD Diabetes patients		AD Prediabetes patients		AD Controls	
	r	p	r	p	r	p
Age (years)	0.04	0.713	0.15	0.582	-0.27	0.115
Body mass index (kg/m^2)	-0.24	0.021	-0.37	0.174	-0.17	0.338
NDS	-0.03	0.764	0.07	0.815	-0.07	0.701
NSS	0.04	0.738	0.22	0.45	-0.06	0.756
HbA1c (%)	0.15	0.162	0.01	0.977	-0.11	0.542
Cystatin C (mg/l)	-0.03	0.783	-0.45	0.147	-0.4	0.026
Glomerular filtration rate (ml/min)	<0.01	0.981	0.45	0.147	0.36	0.05
Triglycerides (mg/dl)	-0.13	0.198	-0.49	0.067	-0.05	0.793
Total serum cholesterol (mg/dl)	0.22	0.037	-0.03	0.934	-0.10	0.574
HDL cholesterol (mg/dl)	0.18	0.079	0.18	0.511	0.08	0.666
LDL cholesterol (mg/dl)	0.16	0.136	0.07	0.803	-0.13	0.445
Sural NCV (m/s)	0.11	0.382	0.31	0.382	0.38	0.027
Sural SNAP (µV)	0.23	0.051	0.25	0.492	0.29	0.098
Peroneal NCV (m/s)	<0.01	0.967	-0.18	0.585	-0.04	0.814
Peroneal CMAP (µV)	-0.09	0.427	0.06	0.854	0.07	0.694
Tibial NCV (m/s)	-0.09	0.397	0.02	0.947	0.18	0.321
Tibial CMAP (µV)	-0.17	0.116	-0.25	0.43	0.09	0.604
Total Pegboard Test Score	-0.01	0.93	-0.41	0.21	0.05	0.82
Pegboard Test of dominant hand	0.06	0.635	-0.59	0.057	0.08	0.695
Pegboard Test of non-dominant hand	-0.06	0.609	-0.31	0.359	-0.13	0.534
Pegboard Test of both hands	0.07	0.569	-0.23	0.49	0.20	0.326
Pegboard Assembly Test	-0.10	0.384	-0.30	0.377	0.15	0.451

NDS = Neuropathy Disability Score; NSS = Neuropathy Severity Score; HbA1c = glycated hemoglobin; GFR = glomerular filtration rate; HDL = high density lipoprotein; LDL = low density lipoprotein; NCV = nerve conduction velocity; m/s = meters per second; SNAP = sensory nerve action potential; CMAP = compound motor action potential; µV = microvolt

Supplementary material, Table S2

Table S2: Correlations of the sciatic nerve's DTI radial diffusivity (RD) with clinical, epidemiological, and serological data of diabetes patients, prediabetes patients, and controls.

	RD Diabetes patients		RD Prediabetes patients		RD Controls	
	r	p	r	p	r	p
Age (years)	0.34	<0.001	0.41	0.131	0.24	0.169
Body mass index (kg/m^2)	-0.07	0.505	-0.14	0.630	-0.08	0.639
NDS	0.35	<0.001	0.41	0.144	0.12	0.495
NSS	0.12	0.239	0.43	0.126	0.25	0.148
HbA1c (%)	0.08	0.416	0.16	0.565	0.23	0.193
Cystatin C (mg/l)	0.43	<0.001	-0.09	0.783	-0.18	0.341
Glomerular filtration rate (ml/min)	-0.4	<0.001	0.09	0.783	0.14	0.448
Triglycerides (mg/dl)	-0.15	0.154	-0.33	0.237	0.05	0.784
Total serum cholesterol (mg/dl)	0.03	0.765	0.02	0.954	0.19	0.262
HDL cholesterol (mg/dl)	0.08	0.469	0.3	0.274	0.15	0.395
LDL cholesterol (mg/dl)	0.02	0.848	0.02	0.954	0.13	0.458
Sural NCV (m/s)	-0.06	0.637	0.54	0.105	0.12	0.489
Sural SNAP (mV)	-0.14	0.234	0.13	0.733	-0.08	0.654
Peroneal NCV (m/s)	-0.43	<0.001	-0.58	0.050	-0.15	0.383
Peroneal CMAP (µV)	-0.58	<0.001	-0.39	0.216	-0.13	0.464
Tibial NCV (m/s)	-0.41	<0.001	-0.22	0.496	-0.13	0.472
Tibial CMAP (µV)	-0.53	<0.001	-0.52	0.089	-0.16	0.368
Total Pegboard Test Score	-0.44	<0.001	-0.73	0.010	-0.54	0.010
Pegboard Test of dominant hand	-0.44	<0.001	-0.83	<0.001	-0.35	0.076
Pegboard Test of non-dominant hand	-0.39	<0.001	-0.67	0.024	-0.48	0.010
Pegboard Test of both hands	-0.29	0.013	-0.63	0.039	-0.35	0.085
Pegboard Assembly Test	-0.43	<0.001	-0.64	0.033	-0.38	0.052

NDS = Neuropathy Disability Score; NSS = Neuropathy Severity Score; HbA1c = glycated hemoglobin; GFR = glomerular filtration rate; HDL = high density lipoprotein; LDL = low density lipoprotein; NCV = nerve conduction velocity; m/s = meters per second; SNAP = sensory nerve action potential; CMAP = compound motor action potential; µV = microvolt

Supplementary material, Table S3

Table S3: Correlations of the sciatic nerve's DTI mean diffusivity (MD) with clinical, epidemiological, and serological data of diabetes patients, prediabetes patients, and controls.

	MD Diabetes patients		MD Prediabetes patients		MD Controls	
	r	p	r	p	r	p
Age (years)	0.23	0.024	0.38	0.159	<0.01	0.983
Body mass index (kg/m^2)	-0.16	0.122	-0.21	0.45	-0.13	0.469
NDS	0.22	0.04	0.27	0.356	0.01	0.941
NSS	0.10	0.319	0.39	0.174	0.1	0.581
HbA1c (%)	0.15	0.153	0.13	0.655	0.09	0.615
Cystatin C (mg/l)	0.22	0.058	-0.17	0.604	-0.29	0.109
Glomerular filtration rate (ml/min)	-0.23	0.048	0.17	0.604	0.25	0.174
Triglycerides (mg/dl)	-0.16	0.133	-0.44	0.106	<0.01	0.994
Total serum cholesterol (mg/dl)	0.14	0.179	0.02	0.944	0.06	0.711
HDL cholesterol (mg/dl)	0.13	0.216	0.31	0.262	0.12	0.475
LDL cholesterol (mg/dl)	0.11	0.292	0.07	0.803	<0.01	0.98
Sural NCV (m/s)	0.01	0.924	0.47	0.171	0.35	0.044
Sural SNAP (mV)	0.03	0.781	0.15	0.682	0.14	0.442
Peroneal NCV (m/s)	-0.27	0.011	-0.46	0.136	-0.07	0.695
Peroneal CMAP (µV)	-0.40	<0.001	-0.24	0.449	-0.01	0.934
Tibial NCV (m/s)	-0.31	0.004	-0.14	0.674	0.04	0.804
Tibial CMAP (µV)	-0.42	<0.001	-0.57	0.059	-0.02	0.927
Total Pegboard Test Score	-0.29	0.010	-0.68	0.020	-0.23	0.260
Pegboard Test of dominant hand	-0.26	0.026	-0.82	0.002	-0.12	0.535
Pegboard Test of non-dominant hand	-0.31	0.008	-0.58	0.062	-0.32	0.109
Pegboard Test of both hands	-0.17	0.155	-0.5	0.118	-0.04	0.859
Pegboard Assembly Test	-0.34	0.003	-0.6	0.053	-0.08	0.983

NDS = Neuropathy Disability Score; NSS = Neuropathy Severity Score; HbA1c = glycated hemoglobin; GFR = glomerular filtration rate; HDL = high density lipoprotein; LDL = low density lipoprotein; NCV = nerve conduction velocity; m/s = meters per second; SNAP = sensory nerve action potential; CMAP = compound motor action potential; µV = microvolt

Supplementary material, Table S4

Table S4: Correlations of the sciatic nerve's DTI eigenvalue λ_2 with clinical, epidemiological, and serological data of diabetes patients, prediabetes patients, and controls.

	λ_2 Diabetes patients		λ_2 Prediabetes patients		λ_2 Controls	
	r	p	r	p	r	p
Age (years)	0.35	0	0.44	0.099	0.26	0.13
Body mass index (kg/m^2)	-0.04	0.713	-0.23	0.419	-0.06	0.752
NDS	0.38	<0.001	0.33	0.249	0.07	0.692
NSS	0.15	0.143	0.46	0.098	0.29	0.097
HbA1c (%)	0.1	0.322	0.14	0.616	0.22	0.201
Cystatin C (mg/l)	0.46	<0.001	-0.17	0.604	-0.2	0.292
Glomerular filtration rate (ml/min)	-0.44	<0.001	0.17	0.604	0.18	0.321
Triglycerides (mg/dl)	-0.15	0.149	-0.39	0.153	-0.01	0.961
Total serum cholesterol (mg/dl)	0.02	0.827	0.01	0.974	0.21	0.229
HDL cholesterol (mg/dl)	0.09	0.414	0.39	0.151	0.18	0.288
LDL cholesterol (mg/dl)	<0.01	0.973	0.01	0.964	0.14	0.431
Sural NCV (m/s)	-0.07	0.601	0.37	0.297	0.14	0.441
Sural SNAP (mV)	-0.18	0.13	0.04	0.918	-0.1	0.573
Peroneal NCV (m/s)	-0.44	<0.001	-0.61	0.037	-0.1	0.578
Peroneal CMAP (µV)	-0.57	<0.001	-0.41	0.182	-0.16	0.369
Tibial NCV (m/s)	-0.4	<0.001	-0.14	0.673	-0.07	0.682
Tibial CMAP (µV)	-0.53	<0.001	-0.52	0.084	-0.18	0.315
Total Pegboard Test Score	-0.43	<0.001	-0.72	0.010	-0.52	0.010
Pegboard Test of dominant hand	-0.44	<0.001	-0.85	0.001	-0.34	0.082
Pegboard Test of non-dominant hand	-0.40	<0.001	-0.67	0.028	-0.56	0.002
Pegboard Test of both hands	-0.28	0.015	-0.61	0.049	-0.32	0.115
Pegboard Assembly Test	-0.43	<0.001	-0.62	0.045	-0.34	0.085

NDS = Neuropathy Disability Score; NSS = Neuropathy Severity Score; HbA1c = glycated hemoglobin; GFR = glomerular filtration rate; HDL = high density lipoprotein; LDL = low density lipoprotein; NCV = nerve conduction velocity; m/s = meters per second; SNAP = sensory nerve action potential; CMAP = compound motor action potential; µV = microvolt

Supplementary material, Table S5

**Table S5:** Correlations of the sciatic nerve's DTI eigenvalue λ_3 with clinical, epidemiological, and serological data of diabetes patients, prediabetes patients, and controls.						
	λ_3 Diabetes patients		λ_3 Prediabetes patients		λ_3 Controls	
	r	p	r	p	r	p
Age (years)	0.31	0.002	0.4	0.141	0.23	0.188
Body mass index (kg/m^2)	-0.1	0.337	-0.04	0.903	-0.07	0.693
NDS	0.32	0.002	0.38	0.185	0.2	0.258
NSS	0.11	0.306	0.36	0.206	0.13	0.47
HbA1c (%)	0.07	0.532	0.17	0.553	0.21	0.223
Cystatin C (mg/l)	0.39	0.001	-0.14	0.667	-0.16	0.399
Glomerular filtration rate (ml/min)	-0.36	0.002	0.14	0.667	0.09	0.625
Triglycerides (mg/dl)	-0.15	0.162	-0.23	0.411	0.11	0.517
Total serum cholesterol (mg/dl)	0.05	0.66	0.10	0.734	0.16	0.354
HDL cholesterol (mg/dl)	0.07	0.482	0.27	0.322	0.07	0.69
LDL cholesterol (mg/dl)	0.04	0.68	0.06	0.822	0.10	0.561
Sural NCV (m/s)	-0.07	0.613	0.52	0.129	0.12	0.507
Sural SNAP (mV)	-0.11	0.343	0.05	0.892	-0.05	0.794
Peroneal NCV (m/s)	-0.41	<0.001	-0.53	0.077	-0.20	0.257
Peroneal CMAP (µV)	-0.56	<0.001	-0.46	0.135	-0.10	0.561
Tibial NCV (m/s)	-0.41	<0.001	-0.19	0.546	-0.14	0.419
Tibial CMAP (µV)	-0.53	<0.001	-0.51	0.094	-0.14	0.435
Total Pegboard Test Score	-0.42	<0.001	-0.71	0.010	-0.49	0.010
Pegboard Test of dominant hand	-0.43	<0.001	-0.71	0.018	-0.29	0.135
Pegboard Test of non-dominant hand	-0.38	0.001	-0.70	0.018	-0.41	0.032
Pegboard Test of both hands	-0.28	0.018	-0.59	0.058	-0.29	0.155
Pegboard Assembly Test	-0.40	<0.001	-0.67	0.028	-0.39	0.045

NDS = Neuropathy Disability Score; NSS = Neuropathy Severity Score; HbA1c = glycated hemoglobin; GFR = glomerular filtration rate; HDL = high density lipoprotein; LDL = low density lipoprotein; NCV = nerve conduction velocity; m/s = meters per second; SNAP = sensory nerve action potential; CMAP = compound motor action potential; µV = microvolt

Supplementary material, Table S6

Table S6: Correlations of the sciatic nerve's FA with results obtained from the Purdue Pegboard Test in male and female diabetes patients, prediabetes patients, and controls.

	FA Diabetes patients		FA Prediabetes patients		FA Controls	
	r	p	r	p	r	p
Total Pegboard Test Score Women	0.71	<0.001	0.93	0.066	0.40	0.115
Pegboard Test of dominant hand Women	0.78	<0.001	0.97	0.031	0.61	0.009
Pegboard Test of non-dominant hand Women	0.58	0.001	0.76	0.237	0.38	0.134
Pegboard Test of both hands Women	0.68	<0.001	0.90	0.103	0.44	0.091
Pegboard Assembly Test Women	0.55	0.003	0.88	0.125	0.53	0.028
Total Pegboard Test Score men	0.46	0.001	0.65	0.111	0.58	0.076
Pegboard Test of dominant hand men	0.42	0.005	0.60	0.154	0.49	0.147
Pegboard Test of non-dominant hand men	0.38	0.009	0.73	0.063	0.70	0.024
Pegboard Test of both hands men	0.21	0.147	0.55	0.199	0.84	0.005
Pegboard Assembly Test men	0.43	0.003	0.55	0.200	0.70	0.024
